# Geographic disparities in hospital readmissions: a retrospective cohort study among patients with chronic disease in rural China

**DOI:** 10.1186/s12939-025-02443-0

**Published:** 2025-03-26

**Authors:** Mingyue Li, Haoqing Tang, Huixian Zheng, Baisong Zhang, Haozhe Cheng, Yanshang Wang, Yuxun Zhou, Xiaotian Zhang, Pascal Geldsetzer, Xiaoyun Liu

**Affiliations:** 1 Department of Health Policy and Management, School of Public Health, Peking University, Beijing, China; 2 China Center for Health Development Studies, Peking University, Beijing, China; 3 Department of Medicine, Division of Primary Care and Population Health, Stanford University, Standford, CA USA; 4 School of Public Health, Imperial College London, London, UK; 5 School of Health Policy & Management, Nanjing Medical University, Nanjing, China; 6 Chan Zuckerberg Biohub, San Francisco, CA USA

**Keywords:** Geographic variation, Hospital readmission, Hypertension, Type 2 diabetes, Rural health

## Abstract

**Background:**

Frequent hospital readmissions place a significant burden on patients, families, and society. Many high-income countries have implemented financial incentives to reduce readmissions. In China, readmission metrics have also been introduced as part of the performance evaluation for secondary hospitals. However, the understanding of hospital readmissions, particularly in rural and remote areas of China, remains limited. This study aims to analyze geographic disparities in hospital readmissions among high-need patients.

**Methods:**

This retrospective cohort study used anonymized hospital discharge data from January 1, 2017, to December 31, 2021, from three public secondary county hospitals. We included rural patients aged 15 and older with hypertension or type 2 diabetes. The outcomes were 30-day, 90-day, and annual readmissions. The explanatory variable was the travel distance to county hospitals, calculated based on the longitude and latitude of registered addresses. Covariates included patient demographics (gender, age, marital status, and ethnicity); health status (Charlson comorbidity score, types of chronic diseases, surgery, and length of stay); and other factors (health insurance and admitted departments). We first reported unweighted readmissions stratified by travel distances (< 40 km versus ≥ 40 km). Multiple logistic regression models were then used to examine the relationship between travel distances and readmissions.

**Results:**

The 30-day, 90-day and annual readmission rates for hypertension or type 2 diabetes were 8.5%, 19.1%, and 39.7%, respectively. Patients living far away were more vulnerable – older (aged 65 and older 59.1% versus 58.5%, *P* < 0.001), predominantly minorities (Minority 55.6% versus 29.4%, P < 0.001), and having more hypertension and diabetes-related complications, as well as undergoing more surgeries (surgery 29.4% versus 23.3%, *P* < 0.001) compared to those living nearby. After adjusting covariates and weights, patients living 40 km away had 11% decrease in the odds of being readmitted within 30 days (OR = 0.89, 95%CI = 0.83–0.96), 10% decrease in the odds of 90-day readmissions (OR = 0.90, 95%CI = 0.85–0.94), and 13% decrease in the odds of annual readmissions (OR = 0.87, 95%CI = 0.84–0.91) compared to those living within 40 km.

**Conclusion:**

We found significant geographic disparities in hospital readmissions among high-need patients. Patients living farther from hospitals had significantly lower odds of readmissions. Readmission rates reflect patients’ healthcare utilization patterns in rural and remote areas. Policymakers should address the geographic access barriers and be cautious when using readmission rates as a measure of hospital performance.

**Supplementary Information:**

The online version contains supplementary material available at 10.1186/s12939-025-02443-0.

## Background

The burden of non-communicable diseases (NCDs) has increased rapidly worldwide, especially in low- and middle-income countries. NCDs are strongly associated with disability and mortality and create tremendous barriers to health equity. 86% of the premature deaths related to NCDs occur in low- and middle-income countries (LMICs). People with NCDs have higher healthcare needs with a higher risk of hospital admissions and readmissions, resulting in high costs, prolonged stays, and a challenging experience navigating the healthcare system. Frequent readmissions usually suggest inadequate quality of care and low health system performance, posing a significant burden to patients, families and society [1] [2] [3]. Therefore, reducing readmissions is a focal goal in improving the quality of care promoting the well-being of patients with NCDs and containing costs.

High-income countries have adopted multiple policies to address readmissions. The Centers for Medicare & Medicaid Services (CMS) launched the Hospital Readmission Reduction Program in the United States in 2012. They introduced penalized strategies for frequent readmissions to improve the quality of the inpatient discharge process and contain costs [4]. England, Denmark, and Germany also collected and released data on readmission rates using different response strategies between 2006 and 2010 [5]. China expanded hospital performance measurement to secondary public hospitals in 2020, alongside financial and non-financial incentives. In 2024, the National Health Commission in China added a readmission rate measure for the first time, focusing on unplanned readmissions among surgical patients as an indicator of hospital care quality [6].

However, our understanding of readmissions, especially in transitional healthcare systems, is far from sufficient to inform policymaking. Readmission is a complex concept affected by multiple factors at the level of individuals, communities, and hospitals [4, 7, 8]. An important yet ambiguous factor is healthcare access [9–13]. Appropriate healthcare access to high-quality care is the foundation for promoting well-being and achieving health equity [14]. In the multifaceted concept of healthcare access, geographic access is the basic dimension, preceding financial access (affordability) and availability [15]. For rural populations, poor geographic access remains a significant challenge [16]. It will result in delays and increased risks of preventable disability or premature death, especially for high-need populations with chronic diseases [17, 18]. Therefore, improving healthcare access for rural populations has consistently been a policy priority on the health agendas of LMICs.

Previous research has provided a conceptual foundation for understanding this issue. A review highlighted that distance is an important factor influencing healthcare utilization decisions among rural and remote populations in countries of the Organisation for Economic Co-operation and Development (OECD) [19]. Some researchers argued that patients’ level of access and readmissions had a bidirectional relationship [20]. While readmission is commonly used as a measure of hospital care quality, it essentially reflects patients’ patterns of inpatient care utilization. We hypothesize that hospital readmission rates at secondary hospitals in rural China will vary according to the distance to the hospital and that the pattern will differ among populations with different healthcare utilization patterns. Our findings will enhance understanding of readmissions in rural and remote areas, and provide implications for other transitional healthcare systems.

## Methods

### Setting and data source

We conducted a retrospective cohort study using anonymized inpatient hospital discharge data from January 1, 2017, to December 31, 2021, collected from three public secondary county hospitals in two underdeveloped provinces in China, Hubei and Henan. This study was approved by the Institute Review Board (IRB) of Peking University Health Science Center (IRB00001052-22155). The data included comprehensive information on diagnoses, medical fees, and basic patient characteristics. The socioeconomic status (SES) in these counties is lower than the national average. In 2022, the annual per capita disposable income of rural residents was $2,006 in County A (Hubei) and $2,166 in County B (Henan), compared to the national average of $2,840. Health resources are also limited, with the number of doctors per thousand population being 2.88 in County A and 2.38 in County B, both below the national average of 2.90 [21].

### Participants

The study included rural inpatients aged 15 and older with chronic diseases of hypertension or type 2 diabetes. Hospital discharge data is composed of admissions, which means that one patient could have multiple admissions in the dataset. Our exclusion criteria were based on admissions. The eligibility criteria were as follows: Rurality was defined in two ways: first, patients reported their jobs as “agricultural workers”; second, patients reported their jobs as “unemployed (no job)” and their living addresses were in villages. This definition is primarily based on economic activity and the place of residence and has been widely used in Chinese studies to define rurality [22]. Diagnoses of primary hypertension (I10, I11, I12, I13, I14) or type 2 diabetes (E11) were based on ICD-10. These diagnoses were identified in the primary admission diagnosis, primary discharge diagnosis, and other secondary discharge diagnoses (the first three diagnoses) in the dataset. For patients transferred from outpatient clinics, their primary outpatient diagnosis was also included for definition.

The exclusion criteria were as follows: 1) significant missing information in primary admission diagnosis, primary discharge diagnosis, or total inpatient fee; 2) transferred from other health facilities; 3) died during hospital; 4) pregnancy-related admissions; 5) planned readmissions: preoperative and postoperative radiotherapy and chemotherapy, pregnancy monitoring, egg retrieval, thawed embryo transfer, suture removal after surgery, follow-up examination, removal of implants, ureteral/drainage tube replacement, rehabilitation therapy, symptomatic treatment after surgery; multiple admissions due to ophthalmic diseases; hemodialysis, peritoneal dialysis, bone marrow transplant, stem cell transplant and inpatient admission due to psychiatric disorders; 6) aged below 15; 7) admitted primarily due to external causes of injury or poisoning.

### Explanatory variables, outcomes, and covariates

The explanatory variable was the travel distance to county hospitals. We obtained the longitude and latitude of addresses using the Application Programming Interface (API) of the Amap application, a mapping service provided by Alibaba Group, one of China's largest technology companies. We then calculated the travel distances to hospitals between the addresses and hospitals. Because we observed a significant drop in readmissions around 40 km, to better interpret the coefficients, we created a dichotomous variable for travel distances above or below 40 km. We also repeated the analyses using travel distance as a continuous variable and by dividing travel distance into five quintiles for sensitivity analyses (appendix).

The outcome variables were hospital readmissions, including 30-day readmission, 90-day readmission, and annual readmission. We identified index discharges and readmissions between January 1, 2017, and December 31, 2021. If a patient had multiple readmissions, only the first readmission following the first index admission was included.

The covariates included: 1) sociodemographics: gender (male vs female), age, marital status (not married including unmarried/divorced/deceased vs married), ethnicity (Han ethnicity vs minorities); 2) health status-related variables: Charlson multimorbidity score, types of chronic diseases (types 2 diabetes, hypertension and comorbid with hypertension and diabetes), whether undergoing surgical procedures (surgery vs none), and length of stay; and 3) other covariates: health insurance (Urban Employee Basic Medical Insurance UEBMI, Urban Residents Basic Medical Insurance URBMI, New Rural Cooperative Medical Scheme NCMS, and other types), and admitted departments (Internal Medicine department, Traditional Chinese Medicine (TCM) department, and other departments). Adjusted covariates also included hospitals (one hospital in County A, and two hospitals in County B) and years of admission (2017, 2018, 2019, 2020, 2021).

### Statistical analysis

We reported and compared the characteristics of patients travelling below and above 40 km to hospitals. To adjust regional variations, logistic regression models or linear regression models were used to examine the statistical significance between the two groups. We used multivariable logistic regression models to analyze the associations between travel distances and readmissions, adjusting for covariates, hospital fixed effects, and time-linear trends. Considering that the population of county A (566.0 thousand) far exceeded that of county B (383.7 thousand) as of 2022, we used county population as weights in all models to correct for overrepresentation. Standard errors were clustered at the patient level to account for the correlations of multiple admissions of each patient. This study adheres to the STROBE checklist for cohort studies. Statistical significance was set at α = 0.05. All analyses were conducted using Stata V.17.0 (Stata Corp LP).

## Results

**Fig. 1:**
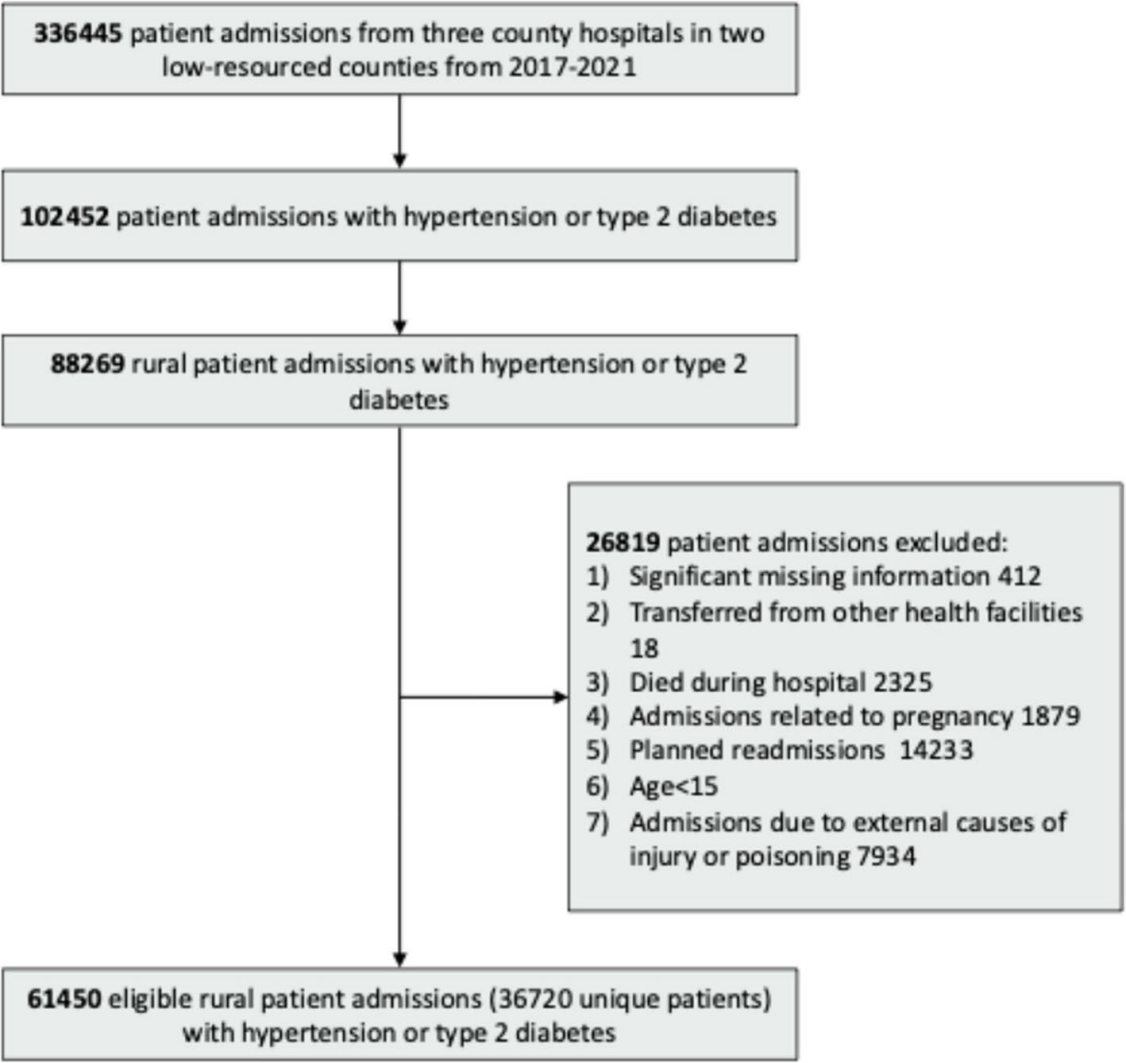
Cohort development of the patients

After exclusions, this study included 61,450 rural patient admissions with hypertension or type 2 diabetes between 2017 and 2021 (Fig. 1). Among these, 41,535 admissions were for patients with hypertension, 8,214 were for patients with type 2 diabetes, and 11,701 were for patients with patients comorbid with hypertension and type 2 diabetes. Compared to the comparative group, admitted patients living more than 40 km from hospitals had a mean age of 65.7 years old, of whom 59.1% were aged 65 and older. They were also comprised of more minority ethnicities (55.6% vs. 29.4%), had more URBMI/NCMS (82.1% vs. 81.0%), and less Medical Assistance as health insurance (8.5% vs. 10.5%) (Table 1).

**Table 1:** Basic characteristics of the patients by travel distance to hospitals, N (column %)

Characteristics	Travel distance to hospitals		
	≤ 40 km	> 40 km	Total
Gender			
Female	25,027 (53.6%)	7725 (52.2%)	32,752 (53.3%)
Male	21,632 (46.4%)	7066 (47.8%)	28,698 (46.7%)
Marriage			
Not married	8969 (19.2%)	2479 (16.8%)	11,448 (18.6%)
Married	37,690 (80.8%)	12,312 (83.2%)	50,002 (81.4%)
Age	66.034 (12.4)	65.705 (11.7)	65.955 (12.3)
Age category			
< 65	19,365 (41.5%)	6052 (40.9%)	25,417 (41.4%)
≥ 65	27,294 (58.5%)	8739 (59.1%)	36,033 (58.6%)
Ethnicity			
Minority	13,697 (29.4%)	8225 (55.6%)	21,922 (35.7%)
Han	32,962 (70.6%)	6566 (44.4%)	39,528 (64.3%)
Health insurance			
UEBMI	3008 (6.4%)	946 (6.4%)	3954 (6.4%)
URBMI/NCMS	37,811 (81.0%)	12,137 (82.1%)	49,948 (81.3%)
Medical Assistance	4905 (10.5%)	1251 (8.5%)	6156 (10.0%)
Other	935 (2.0%)	457 (3.1%)	1392 (2.3%)
Years of admission			
2017	6395 (13.7%)	2125 (14.4%)	8520 (13.9%)
2018	8975 (19.2%)	2849 (19.3%)	11,824 (19.2%)
2019	10,990 (23.6%)	3551 (24.0%)	14,541 (23.7%)
2020	10,138 (21.7%)	3091 (20.9%)	13,229 (21.5%)
2021	10,161 (21.8%)	3175 (21.5%)	13,336 (21.7%)
Charlson comorbidity score	1.390 (1.7)	1.266 (1.6)	1.360 (1.7)
Charlson comorbidity score category			
0	11,253 (24.1%)	4285 (29.0%)	15,538 (25.3%)
1	21,457 (46.0%)	6522 (44.1%)	27,979 (45.5%)
2 or 3	11,404 (24.4%)	3293 (22.3%)	14,697 (23.9%)
> 3	2545 (5.5%)	691 (4.7%)	3236 (5.3%)
Types of chronic diseases			
Hypertension	30,793 (66.0%)	10,742 (72.6%)	41,535 (67.6%)
Diabetes	6408 (13.7%)	1806 (12.2%)	8214 (13.4%)
Comorbid with hypertension and diabetes	9458 (20.3%)	2243 (15.2%)	11,701 (19.0%)

*UEBMI-Urban Employee Basic Medical Insurance, URBMI-Urban Residents Basic Medical Insurance, NCMS-New Rural Cooperative Medical Scheme*

Table 2 presents the results of admissions and readmissions stratified by travel distance. The 30-day readmission rate was 8.5%, the 90-day readmission rate was 19.1%, and the annual readmission rate was 39.7%. Compared to patients living within 40 km, the 30-day readmission rate was 1.2 percentage points lower (*P* = 0.007), the 90-day readmission rate was 1.2 percentage points lower (*P* < 0.001), and the annual readmission rate was 2.3 percentage points lower (*P* < 0.001) for those living beyond 40 km (Table 2). Readmission rates were also significantly higher in the sub-group of women, aged 65 and older, Medical Assistance, no surgery, more comorbidities and admitted to TCM (Table A1, appendix).

**Table 2:** Characteristics of patients’ admissions by travel distance to hospitals

Characteristics	Travel distance to hospitals			Effect size (95% CI)	*P* value
	≤ 40 km	> 40 km	Total		
30-day readmission rate, N(%)	4106 (8.8%)	1118 (7.6%)	5224 (8.5%)	0.91 (0.84—0.98) ^b^	0.007
90-day readmission rate, N(%)	9052 (19.4%)	2699 (18.2%)	11,751 (19.1%)	0.91 (0.87—0.96) ^b^	< 0.001
Annual readmission rate, N(%)	18,773 (40.2%)	5603 (37.9%)	24,376 (39.7%)	0.89 (0.85—0.93) ^b^	< 0.001
Inpatient expenditure, hundred CNY, mean (SD)	6627.5 (7611.4)	6696.5 (7882.8)	6644.1 (7677.6)	2.76% (1.49%−4.04%) ^a^	0.001
Length of stay, mean (SD)	10.014 (7.8)	10.127 (7.7)	10.041 (7.8)	2.04% (0.79%−3.30%) ^a^	< 0.001
Surgery, N(%)	10,884 (23.3%)	4353 (29.4%)	15,237 (24.8%)	1.09 (1.04–1.14) ^b^	< 0.001
Admitted departments, N(%)					
Internal Medicine	26,860 (57.6%)	7633 (51.6%)	34,493 (56.1%)	Ref	
Other	10,489 (22.5%)	2983 (20.2%)	13,472 (21.9%)	0.09 (0.04–0.14) ^b^	< 0.001
TCM	9310 (20.0%)	4175 (28.2%)	13,485 (21.9%)	0.07 (0.00–0.15) ^b^	< 0.001

*TCM-Traditional Chinese Medicine; CNY-Chinese yuan. Time-linear trends and hospital fixed effects were controlled in all regression models, but not presented due to space limit. *^*a*^* The effect size represents the regression coefficients multiplied by 100. *^*b*^* The effect size represents the Odds Ratio*

Patients living above 40 km seemed to have more complications: a higher proportion of ocular complications (12.5% vs. 11.6%, *P* = 0.049), neurological complications (15.9% vs. 15.5%, *P* = 0.008), peripheral circulatory complications (2.8% vs. 2.0%, *P* = 0.004), hypertensive emergency (8.2% vs. 5.3%, *P* = 0.004), and hypertensive urgency (0.9% vs. 0.4%, *P* = 0.016) (Table A2, appendix). Besides, patients living 40 km away from hospitals spent 2.76% more (6697 CNY vs. 6628 CNY, *P* = 0.001), a longer length of stay (10.127 days vs. 10.014 days, *P* < 0.001), and a substantially higher probability of undergoing surgery (29.4% vs. 23.3%, *P* < 0.001) (Table 2).

**Fig. 2:**
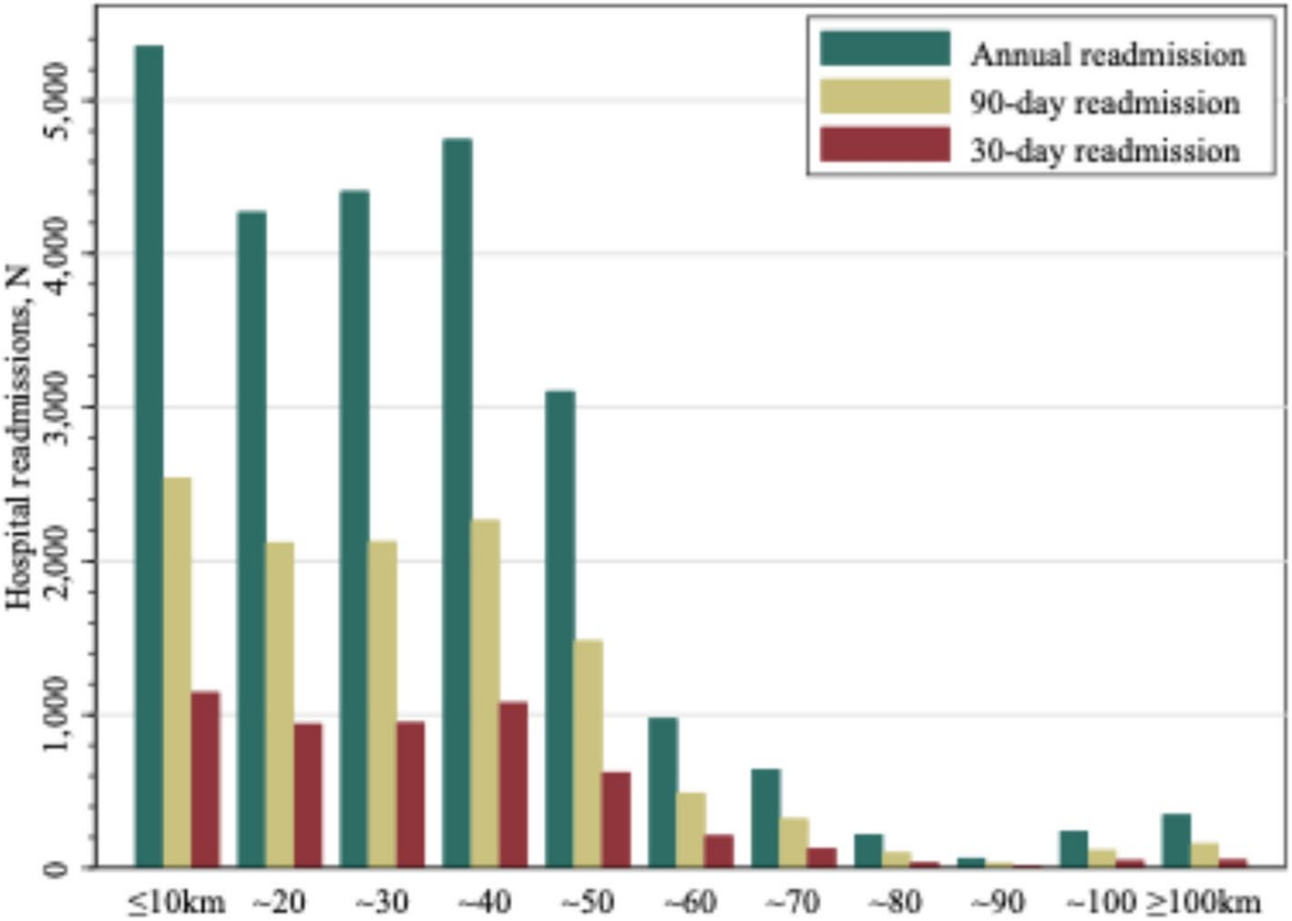
Frequency of hospital readmissions by travel distance to hospitals

Figure 2 displays the distribution of readmissions by travel distance. There was a higher concentration of readmissions among patients living closer, especially within 40 km. Patients living within 10 km exhibited the highest readmission rates, whereas those living farther than 40 km showed substantially lower rates. The pattern was consistent across 30-day, 90-day, and annual readmissions (Fig. 2).

Tables 3 to 4 illustrate the relationships between travel distances and readmissions. Overall, patients living more than 40 km away from hospitals had significantly lower odds of readmission after adjusting for hospital fixed effects, years of admissions, sociodemographic factors, health insurance, admitted departments, and health status. Compared to patients living within 40 km of hospitals, those living farther have a 11% decrease in the odds of 30-day readmissions (OR = 0.89, 95%CI = 0.83–0.96), 10% decrease in the odds of 90-day readmissions (OR = 0.90, 95%CI = 0.85–0.94), and 13% decrease in the odds of annual readmissions (OR = 0.87, 95%CI = 0.84–0.91). These associations remain consistent after stepwise adjustment for confounding variables.

**Table 3:** The associations between travel distances and 30-day hospital readmissions

Characteristics	(1)		(2)		(3)		(4)		(5)	
	OR	95%CI	OR	95%CI	OR	95%CI	OR	95%CI	OR	95%CI
**Geographic access**										
> 40 km (ref = ≤ 40 km)	0.91**	[0.84–0.98]	0.91*	[0.85–0.98]	0.90**	[0.84–0.97]	0.89**	[0.83–0.96]	0.89**	[0.83–0.96]
**Sociodemographic factors**										
Gender (ref = female)			0.98	[0.92–1.04]	0.98	[0.92–1.04]	0.99	[0.94–1.05]	0.98	[0.92–1.04]
Age			1.01***	[1.00–1.01]	1.01***	[1.00–1.01]	1.01***	[1.01–1.01]	1.01***	[1.01–1.01]
Marriage (ref = not married)			1.02	[0.95–1.10]	1.03	[0.95–1.11]	1.03	[0.95–1.11]	1.03	[0.95–1.11]
Ethnicity (ref = Han)			1.10	[0.99–1.23]	1.10	[0.99–1.23]	1.16**	[1.04–1.30]	1.14*	[1.02–1.28]
**Health insurance** (ref = UEBMI)										
URBMI/NCMS					1.21**	[1.06–1.38]	1.22**	[1.07–1.39]	1.23**	[1.08–1.40]
Medical Assistance					1.56***	[1.34–1.82]	1.54***	[1.32–1.80]	1.42***	[1.21–1.66]
Other					1.10	[0.86–1.41]	1.09	[0.85–1.40]	1.06	[0.82–1.37]
**Admitted department** (ref = Internal Medicine)										
Other							1.37***	[1.28–1.47]	1.33***	[1.23–1.43]
TCM							2.84***	[2.56–3.14]	2.39***	[2.16–2.65]
**Health status-related**										
Charlson comorbidity score									1.11***	[1.09–1.13]
Types of chronic disease (ref = hypertension)										
Diabetes									1.02	[0.93–1.12]
Comorbid with hypertension and diabetes									1.06	[0.98–1.14]
Surgery (ref = none)									0.86***	[0.79–0.93]
Length of stay									1.02***	[1.02–1.03]

Time-linear trends and hospital fixed effects were controlled in all regression models but not presented due to space limit. UEBMI-Urban Employee Basic Medical Insurance, URBMI-Urban Residents Basic Medical Insurance, NCMS-New Rural Cooperative Medical Scheme, TCM-traditional Chinese Medicine. Robust 95% confidence interval in brackets; Standard errors were clustered at the patient level. *** p < 0.001, ** p < 0.01, * p < *0.05*

For 30-day readmissions, sociodemographic factors and health status-related variables did not affect the association between travel distance and readmissions. However, health insurance and admitted departments were significant confounding factors in this relationship. Patients more than 40 km away experienced 10% decrease in the odds of 30-day readmissions when health insurance was included in the model (OR = 0.90, 95%CI = 0.84–0.97). This reduction increased to 11% decrease in the odds of readmissions when the admitted departments were also included in the model (OR = 0.89, 95%CI = 0.83–0.96) (Table 3).

For 90-day readmissions, patients living more than 40 km away had 9% decrease in the odds of readmissions (OR = 0.91, 95%CI = 0.87–0.96) in model 1. This association decreased to 8% (OR = 0.92, 95%CI = 0.86–0.95) after including sociodemographic factors, indicating that these factors partially explained the relationship between travel distance and 90-day readmissions. Health insurance was another significant confounding factor; the effect size for travel distance increased from 8% (OR = 0.92, 95%CI = 0.86–0.95) to 10% (OR = 0.90, 95%CI = 0.86–0.95) decrease in the odds of being readmitted. Admitted departments and health status-related variables did not affect the association between travel distance and 90-day readmissions (Table 4).

**Table 4:** The associations between travel distances and 90-day hospital readmissions

Characteristics	(1)		(2)		(3)		(4)		(5)	
	OR	95%CI	OR	95%CI	OR	95%CI	OR	95%CI	OR	95%CI
**Geographic access**										
> 40 km (ref = ≤ 40 km)	0.91***	[0.87–0.96]	0.92**	[0.88–0.97]	0.90***	[0.86–0.95]	0.90***	[0.85–0.94]	0.90***	[0.85–0.94]
**Sociodemographic factors**										
Gender (ref = female)			0.95*	[0.91–0.99]	0.95*	[0.91–0.99]	0.95*	[0.91–0.99]	0.94**	[0.90–0.98]
Age			1.01***	[1.01–1.01]	1.01***	[1.01–1.01]	1.01***	[1.01–1.02]	1.01***	[1.01–1.01]
Marriage (ref = not married)			0.98	[0.93–1.04]	0.99	[0.94–1.05]	0.99	[0.94–1.05]	0.99	[0.93–1.04]
Ethnicity (ref = Han)			1.01	[0.93–1.08]	1.01	[0.93–1.08]	1.03	[0.96–1.11]	1.01	[0.94–1.09]
**Health insurance** (ref = UEBMI)										
URBMI/NCMS					1.15**	[1.06–1.26]	1.16***	[1.06–1.27]	1.18***	[1.08–1.29]
Medical Assistance					1.79***	[1.61–2.00]	1.79***	[1.60–1.99]	1.66***	[1.48–1.85]
Other					0.97	[0.81–1.15]	0.97	[0.81–1.15]	0.95	[0.79–1.13]
**Admitted department** (ref = Internal Medicine)										
Other							1.15***	[1.09–1.21]	1.12***	[1.05–1.18]
TCM							2.07***	[1.91–2.23]	1.81***	[1.67–1.96]
**Health status-related**										
Charlson comorbidity score									1.14***	[1.13–1.15]
Types of chronic disease (ref = hypertension)										
Diabetes									1.07*	[1.00–1.14]
Comorbid with hypertension and diabetes									1.07*	[1.02–1.14]
Surgery (ref = none)									0.85***	[0.80–0.90]
Length of stay									1.03***	[1.02–1.03]

Time-linear trends and hospital fixed effects were controlled in all regression models, but not presented due to space limit. UEBMI-Urban Employee Basic Medical Insurance, URBMI-Urban Residents Basic Medical Insurance, NCMS-New Rural Cooperative Medical Scheme, TCM-traditional Chinese Medicine. Robust 95% confidence interval in brackets; Standard errors were clustered at the patient level. *** p < 0.001, ** p < 0.01, * p < *0.05*

For annual readmissions, patients living more than 40 km away had 11% decrease in the odds of readmissions (OR = 0.89, 95%CI = 0.85–0.93) in model 1. This association decreased to 10% (OR = 0.90, 95%CI = 0.86–0.94) after including sociodemographic factors, suggesting that these factors partially explain the relationship between travel distance and annual readmissions. Health insurance was another significant confounding factor: the effect size for travel distance increased from 10% (OR = 0.90, 95%CI = 0.86–0.94) to 13% (OR = 0.87, 95%CI = 0.83–0.91) decrease in the odds of being readmitted. Admitted departments and health status-related variables did not affect the association between travel distance and annual readmissions (Table A3).

Patients with URBMI/NCMS or Medical Assistance have significantly higher odds of all readmissions. Particularly for Medical Assistance, patients have 1.56 times higher odds of 30-day readmissions (95%CI = 1.34–1.82) (Table 3), 1.79 times higher odds of 90-day readmissions (95%CI = 1.61–2.00) (Table 4), and 2.38 times higher odds of annual readmissions (95%CI = 2.18–2.61) (Table A3).

**Fig. 3:**
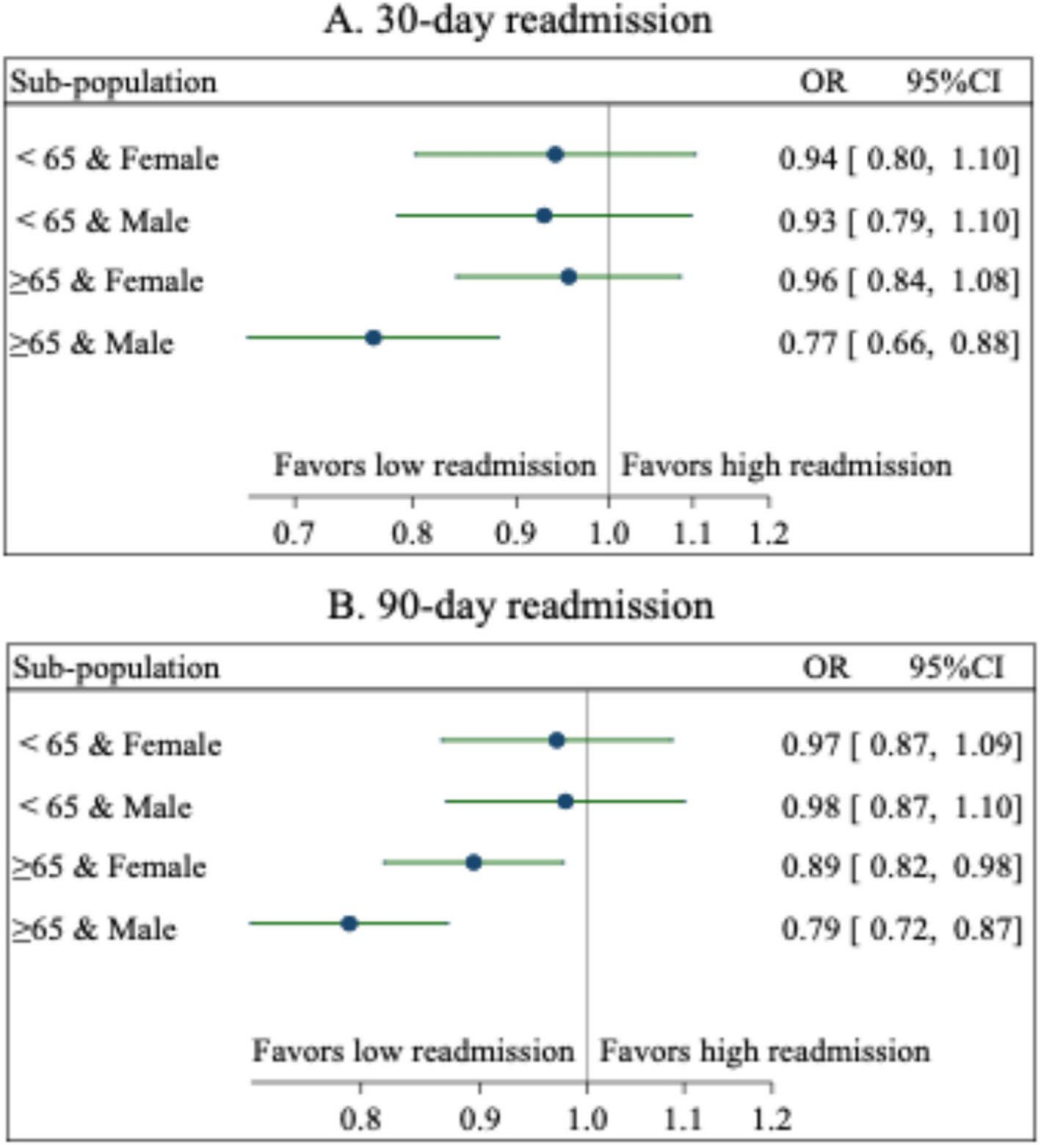
The associations between travel distance and readmissions among population subgroups

Given that sociodemographic factors explained the association between travel distance and 90-day/annual readmissions, and that age and gender were significant influencing factors for readmissions, we conducted subgroup analyses by gender and age category. Figure 3 shows that the association between travel distance and readmission was most pronounced in patients aged 65 years and older. For 30-day readmission, living more than 40 km from the hospital lowered the odds of 30-day readmission by 23% (OR = 0.77, 95% CI = 0.66–0.88) in male patients aged 65 years and older, odds of 90-day readmission by 21% (OR = 0.79, 95% CI = 0.72–0.87), and odds of annual readmission by 20% (Fig. A1, OR = 0.80, 95% CI = 0.74–0.87). For female patients aged 65 years and older, travel distance above 40 km significantly lowered their odds of 90-day readmissions (OR = 0.89, 95% CI = 0.82–0.98) and annual readmissions (Fig. A1, OR = 0.86, 95% CI = 0.80–0.93) (Fig. 3).

## Discussion

This study examined the geographic disparities in hospital readmissions among patients with hypertension or type 2 diabetes in rural China. Our findings contribute to the existing studies in several ways. First, we identified significant disparities in readmission rates at secondary county hospitals: patients living farther from hospitals had substantially lower odds of readmission, with 11% fewer 30-day readmissions, 10% fewer 90-day readmissions, and 13% fewer annual readmissions. Despite these lower readmission rates, these patients are more vulnerable, as they tend to be older, predominantly from minority groups, and experience higher rates of hypertension- or diabetes-related complications, along with undergoing more surgeries than those living closer to hospitals. Second, gender and age emerged as key factors influencing readmission disparities. Patients residing more than 40 km away are more likely to be male and aged 65 or older, which adds to the challenges in accessing healthcare. Third, health insurance, a significant factor contributing to readmissions, mitigated some of the barriers related to geographic access.

Previous research showed that the association between travel distance and readmissions may vary depending on the specific chronic conditions and types of care received. As early as 2000, longer distances reduced healthcare utilization among the rural elderly in the UK, because location represented “a richer web of relations between residents and their local communities” [23]. Studies from the US also found that 90-day readmission rates were significantly lower for patients living farther away (OR = 0.97 for an increase of 100 km, 95%CI = 0.96–0.98), probably due to poorer access [7]. Readmissions were lower in Medicare patients in remote rural areas (OR = 0.74, 95%CI = 0.57–0.95), probably due to lower access to follow-up care and a lack of interactions with physicians [24]. At the same time, urban hospitals had higher odds of 30-day readmissions across multiple conditions compared to rural hospitals, likely due to social determinants of post-discharge care access including financial affordability, lack of transportation, poor health literacy, and self-care regimens [25]. In Italy, substantial geographic variations in 30-day readmissions were also documented [12]. In China, a study on psychiatric patients found urban patients had significantly higher odds of readmission compared to rural ones [26].

Several mechanisms within and beyond the healthcare system could explain the geographic disparities in readmissions in rural China. First, longer travel distances reduce geographical access to healthcare, as it can be logistically complex and exhausting for patients in remote rural areas, thereby leading to lower readmissions. Poor geographical access can result from various factors, including limited private transportation, poor road conditions, and inflexible public transportation [27]. These challenges disproportionately affect vulnerable populations. Our finding that patients living farther away are also more vulnerable supports this point. Sub-group analysis further provided support for this, showing that geographic disparities were most pronounced among rural elder patients. For these vulnerable groups, travelling from rural residences to county hospitals for inpatient services is particularly challenging. Since these patients are more likely to depend on others for transportation, longer travel distances impose excessive financial and time burdens on their caregivers, who are often key labourers in the households.

Second, longer travel distances may be a surrogate for poorer individual-level SES and poorer community-level SES. In China, county hospitals are predominantly clustered in more developed downtown areas. Previous researchers proposed the assumption that China’s concentrated distribution of county hospitals likely contributes to the differential utilization of inpatient services [28]. Our findings provide evidence for this assumption. A longer travel distance to hospitals suggests that these patients are more likely to live in underdeveloped areas with relatively lower SES, particularly in western China. In these areas, underutilization is common due to poor access, low service coverage, low health literacy, and non-adherence [29]. Our results align with broader patterns of the underutilization of inpatient services among vulnerable populations [30]. Although we accounted for various individual-level sociodemographic factors such as gender, age, marital status, and ethnicity, we were unable to obtain SES due to data limitations.

While poor geographic access makes it more difficult for rural residents to be readmitted to hospitals, health insurance helps mitigate this challenge. China has established a national health insurance system, supplemented by targeted insurance and private insurance and achieved universal coverage by 2011 [31, 32]. Poverty alleviation initiatives and other support measures for vulnerable groups have also significantly increased inpatient utilization [33]. According to China’s national health survey, the inpatient rate in rural areas has risen substantially, with an age-standardized annual growth rate of 13.6% [34], largely due to the expansion of insurance coverage [35]. Our findings are consistent with previous evidence on the impact of health insurance. Health insurance is strongly associated with increased readmissions, especially for Medical Assistance, which targets the most impoverished populations. However, even after adjusting for insurance, longer travel distances remain significantly associated with lower readmission rates. Thus, health insurance cannot fully overcome geographical barriers. For vulnerable groups living in remote areas, health insurance encourages the use of inpatient services, but geographic access is still a significant challenge.

Deficiencies in the health system design may exacerbate the problem. China’s health insurance provides a higher reimbursement rate for inpatient services than for outpatient services, incentivizing both hospitals and patients to favour inpatient care. This has been recognized as a major contributor to the rising inpatient rates in China [36]. While underutilization is easier to detect based on patients’ health status, overutilization is more challenging to trace. This difficulty arises from the need for subjective assessments of the appropriateness of admissions, which are often coupled with uncertainty – a universal challenge. A systematic review examining geographic variations in the overuse of specific procedures in the US reached a mixed conclusion [37].

This study has several implications for future research and policy development. First, stakeholders—including policymakers, local health workers, and hospital leaders—must collaborate to reduce geographic barriers for individuals living in remote areas. Enhanced transportation options have been found to increase healthcare utilization, especially among poorer and more vulnerable populations [38, 39]. Additionally, the rapid expansion of Internet healthcare services also has the potential for improving access. Second, policymakers and researchers should be cautious when using the readmission rate as a measure of hospital care, especially in the context of transitional healthcare systems. Our results indicate that readmissions exhibit significant geographic disparities and reflect more of patients’ utilization patterns, even for short-term readmissions. In rural China, the healthcare system is still in a transitional phase, with ongoing reforms such as restructuring the service delivery systems, integrating health information systems, integrating health insurance schemes and other reforms [40]. These systematic factors greatly influence patients' healthcare utilization. Therefore, using readmission rate as a measure of hospital performance should be cautious, as patients’ healthcare utilization patterns must be considered. Third, future studies could establish an appropriate benchmark for assessing readmission rates while considering the different stages of socioeconomic development, especially among vulnerable populations. This will provide valuable evidence for the targeted redesign of the rural healthcare system.

This study has several limitations. First, it is challenging to establish a benchmark for readmission rates of rural patients in China now. While we found the geographic disparities in readmissions, we cannot determine whether these represent underreadmissions or overreadmissions due to a lack of an appropriate benchmark. Second, this is a descriptive study, and we cannot determine if living further away causes lower readmission rates. The association between travel distance and readmission could be confounded by unobserved SES factors, such as household income. This study was conducted in secondary county hospitals located in low-resource areas of China, and thus, our findings may not be interpreted as representative of the entire country. Third, we used travel distance to hospitals as a measure of geographic access, without accounting for different modes of transportation. However, given that our study population is restricted to low-resource rural patients with hypertension and diabetes, the low heterogeneity in this population may help mitigate this issue. Lastly, the outbreak of COVID-19may distort the association between travel distance and readmissions. Regional lockdowns may further decrease readmissions for people living far away. Although we have controlled for time-linear trends, such effect may still exist.

## Conclusion

This study examined the geographic disparities in hospital readmissions among rural patients with chronic diseases in China. We found that patients living farther from hospitals had significantly lower odds of readmissions. Policymakers should address the geographic access barriers faced by these rural remote populations. Readmission rates reflect more on patients’ healthcare utilization patterns. Policymakers should be cautious when using and interpreting readmission rates as a measure of hospital performance.

## Supplementary Information

Supplementary Material 1.

## Data Availability

No datasets were generated or analysed during the current study.
