# Supplementary material for: Geographic disparities in hospital readmissions: a retrospective cohort study among patients with chronic disease in rural China
Source: Int J Equity Health. 2025 Mar 26;24:83. doi: 10.1186/s12939-025-02443-0 (PMC11948674; doi:10.1186/s12939-025-02443-0)
Supplement: Supplementary file 1 — Supplementary Material 1. [file 12939_2025_2443_MOESM1_ESM.docx]

**Geographic Disparities in Hospital Readmissions: a Retrospective Cohort Study among Patients with Chronic Disease in Rural China**

**Appendix**

Table A1 Readmission rates of different patient subgroups

| Characteristics | 30-day readmission | | |  | 90-day readmission | | |  | Annual readmission | | |
| --- | --- | --- | --- | --- | --- | --- | --- | --- | --- | --- | --- |
|  | Mean | 95% CI | P value |  | Mean | 95% CI | P value |  | Mean | 95% CI | P value |
| Total | 8.50% | (8.28%-8.72%) | / |  | 19.12% | (18.81%-19.43%) | / |  | 39.67% | (39.28%-40.05%) | / |
| Gender |  |  |  |  |  |  |  |  |  |  |  |
| Female | 8.65% | (8.34%-8.96%) | 0.318 |  | 19.53% | (19.10%-19.96%) | 0.005 |  | 41.29% | (40.76%-41.82%) | <0.001 |
| Male | 8.33% | (8.02%-8.64%) |  |  | 18.66% | (18.21%-19.11%) |  |  | 37.82% | (37.25%-38.39%) |  |
| Marriage |  |  |  |  |  |  |  |  |  |  |  |
| Not married | 8.96% | (8.43%-9.49%) | 0.271 |  | 20.51% | (19.77%-21.25%) | <0.001 |  | 42.53% | (41.63%-43.43%) | <0.001 |
| Married | 8.40% | (8.16%-8.64%) |  |  | 18.81% | (18.48%-19.14%) |  |  | 39.01% | (38.58%-39.44%) |  |
| Age category |  |  |  |  |  |  |  |  |  |  |  |
| ＜65 | 7.92% | (7.59%-8.25%) | <0.001 |  | 17.02% | (16.55%-17.49%) | <0.001 |  | 34.21% | (33.62%-34.80%) | <0.001 |
| ≥65 | 8.91% | (8.62%-9.20%) |  |  | 20.61% | (20.20%-21.02%) |  |  | 43.52% | (43.01%-44.03%) |  |
| Ethnicity |  |  |  |  |  |  |  |  |  |  |  |
| Minority | 7.41% | (7.06%-7.76%) | 0.230 |  | 19.46% | (18.93%-19.99%) | 0.771 |  | 40.44% | (39.79%-41.09%) | 0.142 |
| Han | 9.10% | (8.83%-9.37%) |  |  | 18.94% | (18.55%-19.33%) |  |  | 39.24% | (38.75%-39.73%) |  |
| Health insurance |  |  |  |  |  |  |  |  |  |  |  |
| UEBMI | 7.31% | (6.51%-8.11%) | ref. |  | 18.41% | (17.19%-19.63%) | ref. |  | 38.64% | (37.13%-40.15%) | ref. |
| URBMI/NCMS | 8.24% | (8.00%-8.48%) | 0.181 |  | 18.52% | (18.19%-18.85%) | 0.327 |  | 38.35% | (37.92%-38.78%) | 0.317 |
| Medical assistance | 11.73% | (10.93% -12.53%) | <0.001 |  | 25.24% | (24.16% -26.32%) | <0.001 |  | 53.23% | (51.98% -54.48%) | <0.001 |
| Other | 6.97% | (5.64% -8.30%) | 0.673 |  | 15.59% | (13.69% -17.49%) | 0.018 |  | 29.96% | (27.55% -32.37%) | <0.001 |
| Surgery |  |  |  |  |  |  |  |  |  |  |  |
| No | 8.87% | (8.62%-9.12%) | <0.001 |  | 19.69% | (19.34%-20.04%) | <0.001 |  | 41.01% | (40.56%-41.46%) | <0.001 |
| Yes | 7.37% | (6.96%-7.78%) |  |  | 17.41% | (16.80%-18.02%) |  |  | 35.61% | (34.85%-36.37%) |  |
| Years of admission |  |  |  |  |  |  |  |  |  |  |  |
| 2017 | 8.33% | (7.74%-8.92%) | ref. |  | 17.46% | (16.66%-18.26%) | ref. |  | 27.62% | (26.68%-28.56%) | ref. |
| 2018 | 9.18% | (8.65%-9.71%) | 0.063 |  | 19.79% | (19.06%-20.52%) | <0.001 |  | 42.32% | (41.44%-43.20%) | <0.001 |
| 2019 | 8.50% | (8.05%-8.95%) | 0.884 |  | 19.99% | (19.34%-20.64%) | <0.001 |  | 42.36% | (41.56%-43.16%) | <0.001 |
| 2020 | 9.40% | (8.91%-9.89%) | 0.040 |  | 19.65% | (18.96%-20.34%) | <0.001 |  | 41.34% | (40.50%-42.18%) | <0.001 |
| 2021 | 7.12% | (6.69%-7.55%) | <0.001 |  | 18.12% | (17.47%-18.77%) | 0.174 |  | 40.42% | (39.60%-41.24%) | <0.001 |
| Charlson comorbidity score category |  |  |  |  |  |  |  |  |  |  |  |
| 0 | 6.81% | (6.42%-7.20%) | ref. |  | 15.95% | (15.38%-16.52%) | ref. |  | 33.47% | (32.73%-34.21%) | ref. |
| 1 | 8.12% | (7.81%-8.43%) | <0.001 |  | 17.75% | (17.30%-18.20%) | <0.001 |  | 38.51% | (37.94%-39.08%) | <0.001 |
| 2 or 3 | 9.69% | (9.22%-10.16%) | <0.001 |  | 22.17% | (21.50%-22.84%) | <0.001 |  | 45.36% | (44.56%-46.16%) | <0.001 |
| >3 | 14.52% | (13.30%-15.74%) | <0.001 |  | 32.35% | (30.74%-33.96%) | <0.001 |  | 53.58% | (51.86%-55.30%) | <0.001 |
| Types of chronic diseases |  |  |  |  |  |  |  |  |  |  |  |
| Hypertension | 8.35% | (8.08%-8.62%) | ref. |  | 18.71% | (18.34%-19.08%) | ref. |  | 38.63% | (38.16%-39.10%) | ref. |
| Diabetes | 8.19% | (7.60%-8.78%) | 0.307 |  | 18.91% | (18.07%-19.75%) | 0.568 |  | 38.00% | (36.94%-39.06%) | 0.413 |
| Comorbid with hypertension and diabetes | 9.24% | (8.71%-9.77%) | 0.047 |  | 20.72% | (19.99%-21.45%) | <0.001 |  | 44.51% | (43.61%-45.41%) | <0.001 |
| Admitted departments |  |  |  |  |  |  |  |  |  |  |  |
| Internal Medicine | 7.30% | (7.03%-7.57%) | ref. |  | 17.60% | (17.19%-18.01%) | ref. |  | 39.77% | (39.26%-40.28%) | ref. |
| Other | 9.38% | (8.89%-9.87%) | <0.001 |  | 18.68% | (18.01%-19.35%) | 0.012 |  | 35.16% | (34.36%-35.96%) | <0.001 |
| TCM | 10.71% | (10.18%-11.24%) | <0.001 |  | 23.47% | (22.76%-24.18%) | <0.001 |  | 43.92% | (43.08%-44.76%) | <0.001 |

Table A2 Hypertension and type 2 diabetes-related complications by travel distance to hospitals

| Complications | Travel distance to hospitals, N (proportion of patients with complications%) | | |  |
| --- | --- | --- | --- | --- |
|  | ≤40km | ＞40km | Total | P value |
| **Patients with type 2 diabetes** |  |  |  |  |
| Type 2 diabetes with coma | 158 (1.0%) | 28 (0.7%) | 186 (0.9%) | 0.533 |
| Type 2 diabetes with ketoacidosis | 432 (2.7%) | 127 (3.1%) | 559 (2.8%) | 0.191 |
| Type 2 diabetes with renal complications | 1854 (11.7%) | 529 (13.1%) | 2383 (12.0%) | 0.978 |
| Type 2 diabetes with ocular complications | 1841 (11.6%) | 506 (12.5%) | 2347 (11.8%) | **0.049** |
| Type 2 diabetes with neurological complications | 2456 (15.5%) | 642 (15.9%) | 3098 (15.6%) | **0.008** |
| Type 2 diabetes with peripheral circulatory complications | 315 (2.0%) | 114 (2.8%) | 429 (2.2%) | **0.004** |
| Type 2 diabetes with other specified complications | 159 (1.0%) | 51 (1.3%) | 210 (1.1%) | 0.174 |
| Type 2 diabetes with multiple complications | 1512 (9.5%) | 624 (15.4%) | 2136 (10.7%) | 0.178 |
| Type 2 diabetes with unspecified complications | 137 (0.9%) | 69 (1.7%) | 206 (1.0%) | 0.108 |
| **Patients with hypertension** |  |  |  |  |
| Hypertensive heart disease | 2123 (5.3%) | 1062 (8.2%) | 3185 (6.0%) | **0.004** |
| Hypertensive kidney disease | 176 (0.4%) | 120 (0.9%) | 296 (0.6%) | **0.016** |
| Hypertensive heart and kidney disease | 64 (0.2%) | 46 (0.4%) | 110 (0.2%) | 0.213 |
| Hypertensive emergency | 19828 (49.3%) | 5431 (41.8%) | 25259 (47.4%) | 0.79 |
| Hypertensive urgency | 1240 (3.1%) | 429 (3.3%) | 1669 (3.1%) | 0.708 |

Notes: Poisson regression models were used to examine statistical differences between ≤40km and >40km, with counties adjusted.

Table A3 The associations between travel distances and annual hospital readmissions

| Characteristics | (1) | | (2) | | (3) | | (4) | | (5) | |
| --- | --- | --- | --- | --- | --- | --- | --- | --- | --- | --- |
|  | OR | 95%CI | OR | 95%CI | OR | 95%CI | OR | 95%CI | OR | 95%CI |
| **Geographic access** |  |  |  |  |  |  |  |  |  |  |
| ＞40km (ref=≤40km) | 0.89*** | [0.85-0.93] | 0.90*** | [0.86-0.94] | 0.87*** | [0.83-0.91] | 0.87*** | [0.83-0.91] | 0.87*** | [0.84-0.91] |
| **Sociodemogrpahic factors** |  |  |  |  |  |  |  |  |  |  |
| Gender (ref=female) |  |  | 0.87*** | [0.84-0.90] | 0.87*** | [0.84-0.90] | 0.87*** | [0.84-0.90] | 0.87*** | [0.84-0.90] |
| Age |  |  | 1.02*** | [1.02-1.02] | 1.02*** | [1.02-1.02] | 1.02*** | [1.02-1.02] | 1.02*** | [1.02-1.02] |
| Marriage (ref=not married) |  |  | 1.02 | [0.97-1.06] | 1.03 | [0.99-1.08] | 1.03 | [0.98-1.07] | 1.02 | [0.98-1.07] |
| Ethnicity (ref=Han) |  |  | 0.94 | [0.89-1.00] | 0.94 | [0.89-1.00] | 0.93* | [0.88-0.99] | 0.92** | [0.86-0.98] |
| **Health insurance** (ref=UEBMI） | | | | | | | | | | |
| URBMI/NCMS |  |  |  |  | 1.21*** | [1.13-1.30] | 1.21*** | [1.13-1.30] | 1.22*** | [1.14-1.32] |
| Medical Assistance |  |  |  |  | 2.38*** | [2.18-2.61] | 2.37*** | [2.17-2.60] | 2.26*** | [2.06-2.48] |
| Other |  |  |  |  | 0.89 | [0.77-1.02] | 0.89 | [0.77-1.02] | 0.87 | [0.76-1.00] |
| **Admitted department** (ref=Internal Medicine） | | | | | | | | | | |
| Other |  |  |  |  |  |  | 0.87*** | [0.83-0.90] | 0.89*** | [0.85-0.93] |
| TCM |  |  |  |  |  |  | 1.35*** | [1.27-1.44] | 1.26*** | [1.18-1.35] |
| **Health status-related** |  |  |  |  |  |  |  |  |  |  |
| Charlson comorbidity score |  |  |  |  |  |  |  |  | 1.12*** | [1.11-1.14] |
| Types of chronic disease (ref=hypertension) | | | | | | | | | | |
| Diabetes |  |  |  |  |  |  |  |  | 1.05 | [0.99-1.11] |
| Comorbid with hypertension and diabetes |  |  |  |  |  |  |  |  | 1.18*** | [1.13-1.24] |
| Surgery (ref=none) |  |  |  |  |  |  |  |  | 0.78*** | [0.75-0.82] |
| Length of stay |  |  |  |  |  |  |  |  | 1.02*** | [1.02-1.02] |

Notes: Time-linear trends and hospital fixed effects were controlled in all regression models, but not presented due to space limit. UEBMI-Urban Employee Basic Medical Insurance, URBMI-Urban Residents Basic Medical Insurance, NCMS-New Rural Cooperative Medical Scheme, TCM-traditional Chinese Medicine. Robust 95% confidence interval in brackets; Standard errors were clustered at the patient level. *** p<0.001, ** p<0.01, * p<0.05

Figure A1 The associations between travel distance and readmissions among population subgroups

Table A3 The associations between travel distances and hospital readmissions: sensitivity analyses (1)

|  | 30-day readmission | | | 90-day readmission | | | Annual readmission | | |
| --- | --- | --- | --- | --- | --- | --- | --- | --- | --- |
|  | OR | 95% CI | P value | OR | 95% CI | P value | OR | 95% CI | P value |
| **Geographical access** |  |  |  |  |  |  |  |  |  |
| **Quntiles of travel dsitance to hospitals** |  |  |  |  |  |  |  |  |  |
| First quntile (closest) | 1.10 | [1.00-1.21] | 0.055 | 1.12*** | [1.05-1.20] | 0.001 | 1.17*** | [1.11-1.24] | <0.001 |
| Second quintile | 1.14** | [1.04-1.25] | 0.005 | 1.16*** | [1.09-1.24] | <0.001 | 1.18*** | [1.12-1.25] | <0.001 |
| Third quintile | 1.07 | [0.97-1.17] | 0.169 | 1.07 | [1.00-1.14] | 0.064 | 1.11*** | [1.05-1.17] | <0.001 |
| Fourth quintile | 1.17*** | [1.07-1.29] | 0.001 | 1.11** | [1.04-1.19] | 0.002 | 1.11*** | [1.05-1.17] | <0.001 |
| Fifth quintile (farthest) | ref. |  |  | ref. |  |  | ref. |  |  |
| **Sociodemogrpahic factors** |  |  |  |  |  |  |  |  |  |
| Gender (ref=female) | 0.98 | [0.92-1.04] | 0.428 | 0.94** | [0.90-0.98] | 0.003 | 0.87*** | [0.84-0.90] | <0.001 |
| Age | 1.01*** | [1.01-1.01] | <0.001 | 1.01*** | [1.01-1.01] | <0.001 | 1.02*** | [1.02-1.02] | <0.001 |
| Marriage (ref=unmarried and other) | 1.03 | [0.95-1.11] | 0.520 | 0.99 | [0.93-1.04] | 0.650 | 1.02 | [0.98-1.07] | 0.315 |
| Ethnicity (ref=Han) | 1.15* | [1.03-1.29] | 0.012 | 1.02 | [0.94-1.10] | 0.685 | 0.92** | [0.87-0.98] | 0.008 |
| **Health status-related** |  |  |  |  |  |  |  |  |  |
| Charlson comorbidity score | 1.11*** | [1.09-1.13] | <0.001 | 1.14*** | [1.13-1.15] | <0.001 | 1.12*** | [1.11-1.14] | <0.001 |
| Types of chronic disease (ref=hypertension) |  |  |  |  |  |  |  |  |  |
| Diabetes | 1.02 | [0.93-1.12] | 0.654 | 1.07* | [1.00-1.14] | 0.047 | 1.05 | [0.99-1.11] | 0.086 |
| Comorbid with hypertension and diabetes | 1.06 | [0.98-1.14] | 0.153 | 1.07* | [1.02-1.13] | 0.011 | 1.18*** | [1.13-1.23] | <0.001 |
| Surgery (ref=none) | 0.86*** | [0.79-0.93] | <0.001 | 0.85*** | [0.80-0.90] | <0.001 | 0.78*** | [0.75-0.82] | <0.001 |
| Length of stay | 1.02*** | [1.02-1.03] | <0.001 | 1.03*** | [1.02-1.03] | <0.001 | 1.02*** | [1.02-1.02] | <0.001 |
| **Admitted department** (ref=Internal Medicine) | | | | | | | | | |
| Other | 1.33*** | [1.23-1.43] | <0.001 | 1.11*** | [1.05-1.18] | <0.001 | 0.89*** | [0.85-0.93] | <0.001 |
| TCM | 2.39*** | [2.16-2.65] | <0.001 | 1.81*** | [1.67-1.95] | <0.001 | 1.26*** | [1.18-1.35] | <0.001 |
| **Health insurance** (ref=UEBMI) | |  |  |  |  |  |  |  |  |
| URBMI/NCMS | 1.22** | [1.07-1.39] | 0.004 | 1.18*** | [1.07-1.29] | <0.001 | 1.23*** | [1.14-1.32] | <0.001 |
| Medical assistance | 1.40*** | [1.20-1.64] | <0.001 | 1.66*** | [1.49-1.86] | <0.001 | 2.29*** | [2.09-2.51] | <0.001 |
| Other | 1.05 | [0.82-1.36] | 0.694 | 0.95 | [0.79-1.13] | 0.534 | 0.87 | [0.76-1.00] | 0.059 |

Table A4 The associations between travel distances and hospital readmissions: sensitivity analyses (2)

|  | 30-day readmission | | | 90-day readmission | | | Annual readmission | | |
| --- | --- | --- | --- | --- | --- | --- | --- | --- | --- |
|  | OR | 95% CI | P value | OR | 95% CI | P value | OR | 95% CI | P value |
| **Geographical access** |  |  |  |  |  |  |  |  |  |
| Travel dsitance to hospitals (10 km) | 0.98** | [0.97-0.99] | 0.002 | 0.98*** | [0.98-0.99] | <0.001 | 0.98*** | [0.97-0.99] | <0.001 |
| **Sociodemogrpahic factors** |  |  |  |  |  |  |  |  |  |
| Gender (ref=female) | 0.98 | [0.92-1.04] | 0.446 | 0.94** | [0.90-0.98] | 0.003 | 0.87*** | [0.84-0.90] | <0.001 |
| Age | 1.01*** | [1.01-1.01] | <0.001 | 1.01*** | [1.01-1.01] | <0.001 | 1.02*** | [1.02-1.02] | <0.001 |
| Marriage (ref=unmarried and other) | 1.03 | [0.95-1.11] | 0.521 | 0.99 | [0.93-1.04] | 0.653 | 1.02 | [0.98-1.07] | 0.318 |
| Ethnicity (ref=Han) | 1.14* | [1.02-1.28] | 0.018 | 1.01 | [0.94-1.10] | 0.710 | 0.92** | [0.87-0.98] | 0.008 |
| **Health status-related** |  |  |  |  |  |  |  |  |  |
| Charlson comorbidity score | 1.11*** | [1.09-1.13] | <0.001 | 1.14*** | [1.13-1.15] | <0.001 | 1.12*** | [1.11-1.14] | <0.001 |
| Types of chronic disease (ref=hypertension) |  |  |  |  |  |  |  |  |  |
| Diabetes | 1.02 | [0.93-1.12] | 0.654 | 1.07* | [1.00-1.14] | 0.042 | 1.05 | [1.00-1.11] | 0.072 |
| Comorbid with hypertension and diabetes | 1.05 | [0.98-1.14] | 0.170 | 1.07* | [1.02-1.13] | 0.011 | 1.18*** | [1.13-1.24] | <0.001 |
| Surgery (ref=none) | 0.86*** | [0.79-0.93] | <0.001 | 0.85*** | [0.80-0.90] | <0.001 | 0.78*** | [0.75-0.82] | <0.001 |
| Length of stay | 1.02*** | [1.02-1.03] | <0.001 | 1.03*** | [1.02-1.03] | <0.001 | 1.02*** | [1.02-1.02] | <0.001 |
| **Admitted department** (ref=Internal Medicine) |  |  |  |  |  |  |  |  |  |
| Other | 1.33*** | [1.23-1.43] | <0.001 | 1.12*** | [1.05-1.18] | <0.001 | 0.89*** | [0.85-0.93] | <0.001 |
| TCM | 2.39*** | [2.16-2.65] | <0.001 | 1.81*** | [1.67-1.95] | <0.001 | 1.26*** | [1.18-1.35] | <0.001 |
| **Health insurance** (ref=UEBMI) | |  |  |  |  |  |  |  |  |
| URBMI/NCMS | 1.23** | [1.08-1.41] | 0.002 | 1.18*** | [1.08-1.30] | <0.001 | 1.23*** | [1.14-1.32] | <0.001 |
| Medical assistance | 1.43*** | [1.22-1.67] | <0.001 | 1.67*** | [1.49-1.87] | <0.001 | 2.28*** | [2.08-2.49] | <0.001 |
| Other | 1.07 | [0.83-1.38] | 0.599 | 0.96 | [0.80-1.14] | 0.612 | 0.88 | [0.77-1.01] | 0.078 |
